# Recyclability of Plastics from Waste Mobile Phones According to European Union Regulations REACH and RoHS

**DOI:** 10.3390/ma18091979

**Published:** 2025-04-27

**Authors:** Martina Bruno, Silvia Fiore

**Affiliations:** Department of Environment, Land, and Infrastructure Engineering (DIATI), Politecnico di Torino, Corso Duca degli Abruzzi 24, 10129 Turin, Italy; martina.bruno@polito.it

**Keywords:** end-of-life phones, waste from electrical and electronic equipment, plastic waste, flame retardants, potentially toxic elements, recycling

## Abstract

Small waste from electrical and electronic equipment (WEEE) such as waste mobile phones are rich in plastic components. Recycling mobile phones is particularly challenging, since the main interest for recyclers is printed circuit boards, rich in valuable metals, while the plastic components are usually destined for thermal recovery. This study is dedicated to the assessment of the recyclability potential of the plastic fractions of end-of-life (EoL) mobile phones according to the European Union’s (EU) Restriction of Hazardous Substances (RoHS) and Registration, Evaluation, Authorization and Restriction of Chemicals (REACH) directives. A total of 275 plastic items (inventoried as casings, frames, and screens) were dismantled from 100 EoL mobile phones and analyzed to identify the type and abundance of polymers via Fourier-transform infrared spectroscopy (FTIR) and the presence of hazardous elements such as Br, Cl, Pb, and Cd via X-ray fluorescence (XRF). Polycarbonate (PC) (57% of samples) and polymethyl methacrylate (PMMA) (27% of the items) were identified as the most common prevalent polymers. In total, 67% of the items contained Cl (0.84–40,700 mg/kg), and 26% contained Br (0.08–2020 mg/kg). Hg was detected only in one item (17 mg/kg). Cr was found in 17% of the items, with concentrations between 0.37 mg/kg and 915 mg/kg, while Pb was found in 15% of the items in low concentrations (1–90 mg/kg). In conclusion, while hazardous elements are present in the plastic fractions of EoL mobile phones (with higher values in smartphones), their concentrations were below the regulatory limits, suggesting compliance with recycling regulations in the EU.

## 1. Introduction

Waste from electrical and electronic equipment (WEEE) is one of the fastest-growing waste streams worldwide. In 2022, a total of 62 million metric tons (Mt) of WEEE were generated, and the generation rate is expected to exceed 82 Mt/year by 2030 [1]. Regulations targeting WEEE management and recycling are in force in numerous countries in the world, yet most WEEE is improperly managed, with 82.6% classified as “non-documented recycling” and often associated with illegal dumping or improper handling [2]. WEEE recycling is fundamental to reducing environmental pollution, avoiding exposure to hazardous materials due to improper waste management [3], and promoting material circularity by decreasing reliance on mining for the manufacturing of electric and electronic equipment [4].

However, WEEE recycling faces technical and economic challenges. The complex composition of WEEE, which includes metals, plastics, and hazardous substances, complicates recycling processes [5]. Additionally, economic constraints pose a barrier, as the high costs of recycling often lead to a focus on recovering only the most valuable materials, such as printed circuit boards (PCBs) and electronic components, while less profitable materials, such as plastics, are frequently overlooked [5,6]. Despite accounting for 20–30%wt. of WEEE [7], plastic components are often disregarded due to their lower economic value compared to PCBs, even though they significantly contribute to the total volume of waste requiring disposal.

WEEE plastics are made of polymers such as polycarbonate (PC), acrylonitrile–butadiene–styrene (ABS), high-impact polystyrene (HIPS), polyamides (PA), polypropylene (PP), polyethylene (PE), and polyesters, which hold significant potential for recycling or energy recovery [8]. WEEE plastics also contain harmful substances that hinder the recycling process [9]. Conventional recycling of WEEE plastics involves either mechanical, chemical, or thermal processes. Mechanical processes require sorting, shredding, and reprocessing polymers into recycled polymers [8,10,11]. Chemical recycling includes the extraction of hazardous additives such as brominated flame retardants (BFRs) [10,12,13] or pyrolysis and gasification or hydrolysis to break down polymers into their chemical components, which could then be supplied to polymers remanufacturers [10,11]. Thermal processes, instead, involve the incineration of waste plastic for energy recovery [10,11]. These are commonly applied to handle contaminated plastics, and specific treatment of the flue gas is needed to address the release of hazardous pollutants.

Moreover, in the European Union (EU), recycling of WEEE plastics is challenged by the presence of substances regulated by the Restriction of Hazardous Substances (RoHS) Directive [14], such as Pb, Cd, Hg, Cr (VI), polybrominated biphenyls (PBB), and polybrominated diphenyl ethers (PBDE), or listed on the Registration, Evaluation, Authorization and Restriction of Chemicals (REACH) regulation [15]. These substances pose significant environmental and health risks [16], and their removal is essential for safe recycling practices [17]. The limitations on hazardous contaminants in WEEE plastics, as set by regulations such as the RoHS and REACH directives, are crucial for safeguarding human health and preventing pollution [18]. However, while these limitations are necessary for health and environmental protection, they can inadvertently hinder recycling efforts [8,10]. By restricting the presence of certain substances, the recyclability of plastic waste is reduced, and this may limit the overall material recovery [19,20]. Therefore, it is essential to assess and identify the recyclability potential of WEEE plastics to improve the circularity of this sector.

Previous studies have already discussed how the presence of concerning flame retardants, particularly based on Br and Sb, affects the recyclability potential of plastic components from mixed WEEE [21,22,23,24] or from specific waste streams, such as household appliances [25,26,27] or monitors and television screens [26,27]. A recent study suggests that Br concentrations in mixed WEEE are generally below the regulatory limits [22]. Another study found that 18 samples out of the 149 analyzed exceeded these limits, highlighting variability in contamination levels [28]. Overall, a significant variability in Br content was reported in the literature that is also related to specific WEEE categories. Studies on the plastic fractions of televisions and PC monitors and office appliances [26] have consistently found Br concentrations below regulatory thresholds [29,30]. Internet routers have been identified as containing Br levels above the regulatory limits [31], while large household appliances have been found to exceed Sb limits [32]. Eventually, Br concentrations in waste mobile phones generally remain below regulatory limits [33].

Despite approximately 400 M EoL mobile phones being generated globally each year [1] and plastic components accounting for approximately 40%wt. [34], their recyclability potential remains largely underexplored. Previous studies on the recyclability potential of mobile phones’ plastic components primarily focused on polymer characterization [35] and on the quantification of bromine content [33], often overlooking other potentially harmful substances. One study addressed additional elements but analyzed only a limited number of samples [36], underscoring the need for a more comprehensive evaluation of mobile phone plastics. To the best of our knowledge, a study dedicated to the detection of multiple hazardous elements in the plastic components of a statistically significant sample of waste mobile phones has not been published yet. This study aims at contributing to filling this knowledge gap, with the goal of assessing the recyclability potential of the plastic fraction of waste mobile phones according to the hazardous substances and limits defined by the EU regulations REACH and RoHS. In total, 275 plastic items were manually dismantled from 100 EoL mobile phones, inventoried as casings, frames, and screens, and analyzed to identify the type and abundance of polymers via Fourier-transform infrared spectroscopy (FTIR) and the presence of hazardous elements such as Br, Cl, Pb, and Cd via X-ray fluorescence (XRF).

## 2. Materials and Methods

### 2.1. Origin of the Samples

A sample of 100 EoL mobile phones was provided by a WEEE treatment plant in Turin, Italy. The size of the sample set corresponds to the facility’s weekly intake of EoL mobile phones, and the proportion between the number of feature phones (73) and smartphones (27) corresponds to the typical feed composition. The inventory (brand, model, production year) and mass balance (plastic components, metal components, printed circuit boards, and electronic components) of the EoL mobile phones were discussed in a previous study [34].

### 2.2. Recyclability Assessment

The plastic components of the 100 EoL mobile phones were manually separated into single items and then visually analyzed and classified into casings, frames, and screens. The following definitions were applied to identify each item: “casing” refers to the plastic outer shells of the phones, “frame” is the structural internal part, and “screen” is the panel covering the display. Each item was weighed using a KERN PLJ 4200-2F technical balance(Kern & Sohn GmbH, Balingen, Germany).

The material composition and the presence of hazardous substances were analyzed through a Fourier-transform infrared (FTIR) spectrometer (Nicolet™ Summit™ X model from Thermo Scientific™, Thermo Fisher Scientific, Waltham, MA, USA) equipped with an Everest Diamond attenuated total reflectance (ATR) accessory and through a direct excitation energy-dispersive X-ray fluorescence (EDXRF) spectrometer (NEX-DE, Rigaku, Tokyo, Japan vs. model from Rigaku, Rigaku, Tokyo, Japan), equipped with a 60 kV X-ray tube. Casings, frames, and screens were directly placed above the XRF and FTIR sampling windows. FTIR characterization was employed to identify and quantify the primary polymer in the items. Based on XRF results, the weighted average concentration of hazardous elements was calculated by considering both the mass and the concentration of the element in each item within the three categories casings, frames, and screens. For each cell phone type (smartphone or feature phone), the concentrations were weighted according to the mass of each item, and the overall weighted average concentration for the entire sample set was then derived by aggregating the results across all items, categories, and cell phone types. The recyclability potential was assessed by comparison with the concentration thresholds set for flame-retardant additives (Br, Cl, Sb) and potentially toxic elements (PTEs) (Cd, Cd, Hg and Pb) listed in Table 1.

**Figure 1:**
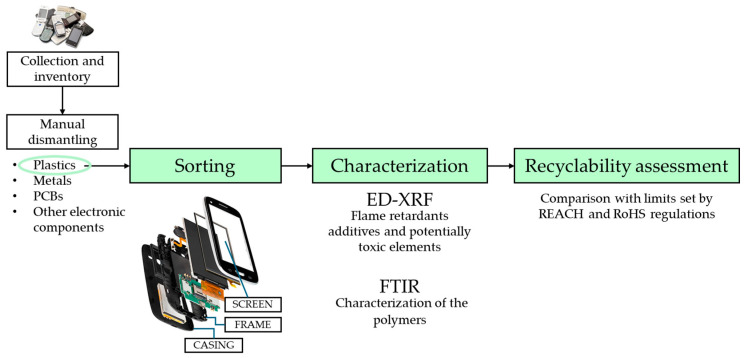
Flowchart of the procedure followed to assess the recyclability of plastic components from EoL mobile phones (PCBs: printed circuit boards; ED-XRF: energy-dispersive X-ray fluorescence spectrometry; FTIR: Fourier-transform infrared spectrometry; REACH: Registration, Evaluation, Authorization and Restriction of Chemicals; RoHS: Restriction of Hazardous Substances).

The procedure followed in this study for sample characterization and recyclability assessment is presented by the flowchart in Figure 1.

## 3. Results

### 3.1. Sample Inventory

**Figure A1:**
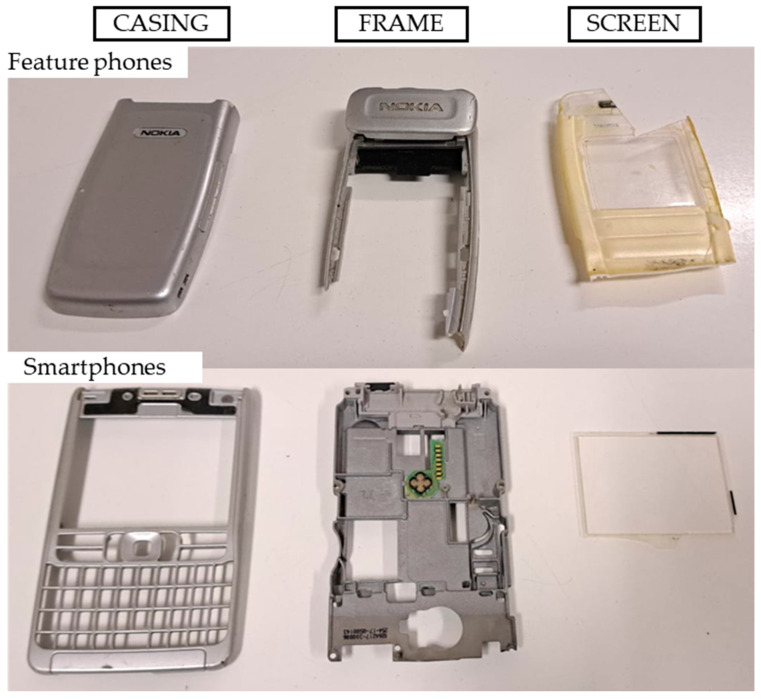
Examples of analyzed components (casings, frames, and screens) from EoL feature phones and smartphones.

The inventory of plastic components from 100 EoL cell phones comprised 275 items, including 73 casings, 73 frames, and 73 screens from EoL feature phones; and 20 casings, 21 frames, and 15 screens from EoL smartphones. Representative examples of the analyzed components are provided in Appendix A, Figure A1. While all the plastic components of the 73 EoL feature phones were collected and inventoried, some plastic components were missing—and therefore excluded from the inventory—from the 27 EoL smartphones (seven casing and six frames), or they were made of nonplastic materials (12 screens identified as glass). This can be ascribed to the smartphones’ design, which makes them more susceptible to damage and loss of parts compared to feature phones.

In both feature phones and smartphones, frames constituted the primary source of plastic, as they are typically large and predominantly composed of plastic materials. Conversely, the plastic contribution from smartphones’ screens was significantly lower, as a substantial share of these was glass-based. Additionally, smartphones’ casings were frequently absent or incomplete due to their multi-component structure, where the outermost layer is commonly removed to facilitate battery removal. Overall, the samples’ set amounted to 3.37 kg of plastic, including 2.58 kg of plastics from feature phones and 0.79 kg from smartphones (Table 2). The manual dismantling and sorting into three categories caused a material loss of 3%wt. compared with the 3.49 kg of the plastic fraction quantified in a previous study [34].

### 3.2. Characterization of the Polymers

**Figure 2:**
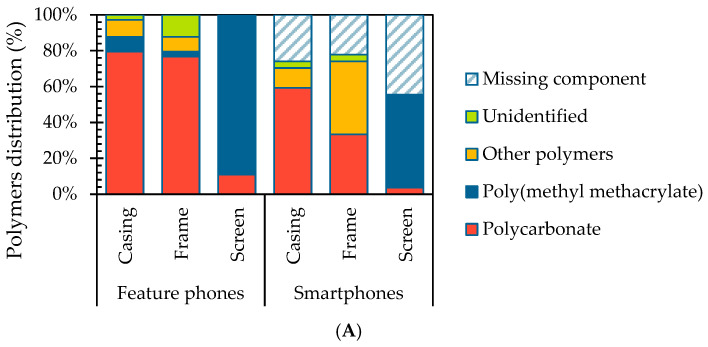

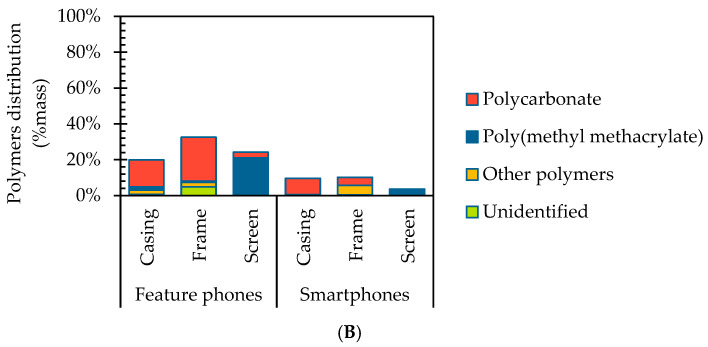
Composition of the plastic items described by (**A**) number of samples and (**B**) mass distribution.

**Figure A2:**
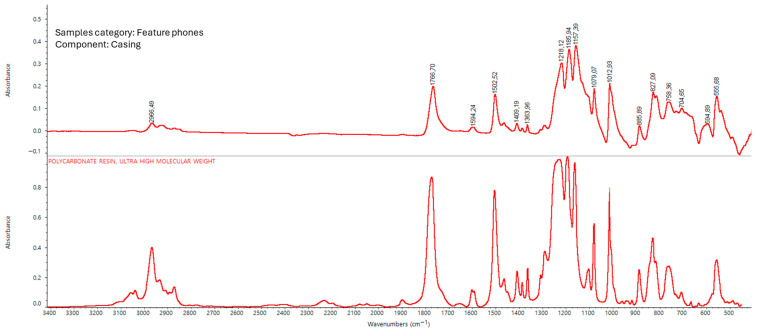
Comparison between the FTIR spectrum acquired from the casing component of a feature phone and the standard FTIR spectrum of polycarbonate.

**Figure A3:**
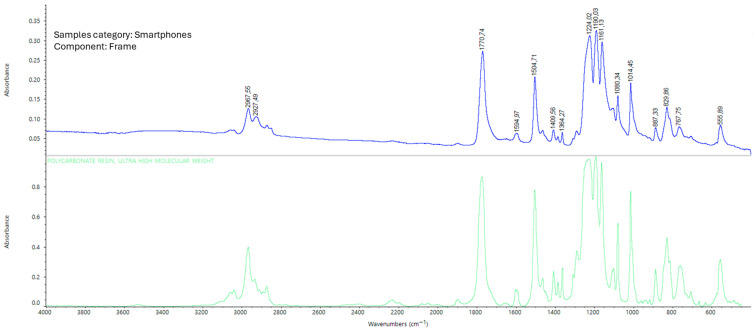
Comparison between the FTIR spectrum acquired from the frame component of a smartphone and the standard FTIR spectrum of polycarbonate.

**Figure A4:**
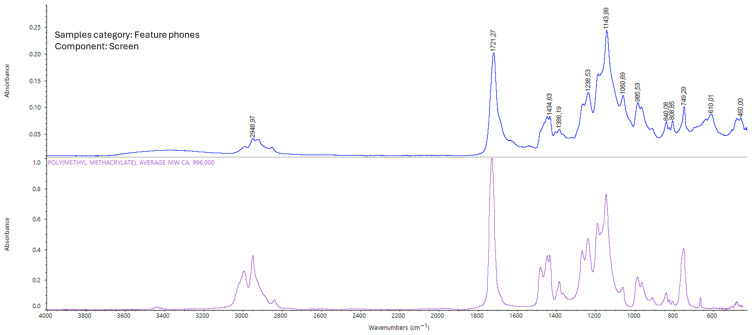
Comparison between the FTIR spectrum acquired from the screen component of a feature phone and the standard FTIR spectrum of polymethyl methacrylate.

The composition of the plastic components was described by identifying and quantifying the polymers composing the items inventoried (Figure 2). Polycarbonate (PC) and polymethyl methacrylate (PMMA) were identified as the most prevalent polymers, with PC accounting for 57% of the items and PMMA for 27% (see Appendix A, Figure A2, Figure A3 and Figure A4). The identification was supported by characteristic FTIR spectra according to the literature: for PC, the spectrum exhibited distinctive peaks at 2967 cm^−1^ corresponding to C–H stretching, 1770 cm^−1^ for C=O stretching, 1504 cm^−1^ for C=C stretching, and at 1189 and 1161 cm^−1^ for C–O stretching [39,40]. In contrast, PMMA showed characteristic peaks at 2948 cm^−1^ for C–H stretching [41], 1721 cm^−1^ for C=O stretching [42], 1434 cm^−1^ for C–H bending, and 1143 cm^−1^ for the C–O ether bond stretching [43]. A similar trend was observed across the device types. In feature phones, plastic components were primarily made of PC (56%) and PMMA (30%), while in smartphones, PC was more common (59%), with a lower presence of PMMA (15%). The remaining polymeric components were clustered as “other polymers”, including acrylonitrile–butadiene–styrene (ABS)/PC blend, polyethylene terephthalate (PET), polystyrene, and poly diallyl phthalate in feature phones. In smartphones, additional polymers such as polyamide and polyethylene-methyl isophthalate were identified in casings and frames from feature phones and in frames from smartphones.

### 3.3. Flame Retardant Additives and Potentially Toxic Elements

**Figure 3:**
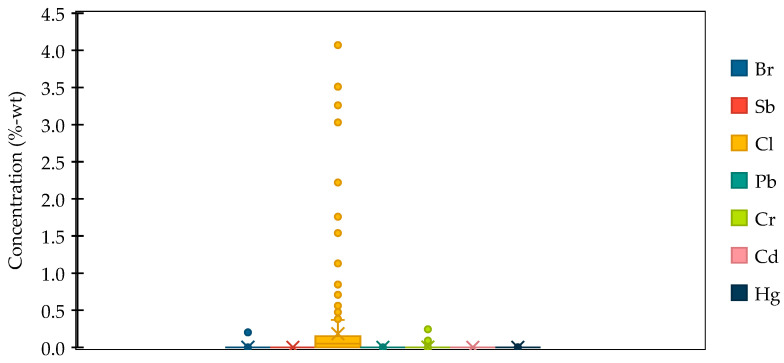
Statistical distribution of the concentrations of flame retardant additives (Br, Sb, Cl) and PTEs (Pb, Cr, Cd, Hg) in the plastic components of EoL cell phones considered in this study.

The detected concentrations of hazardous substances, including flame retardant additives (Br and Cl) and potentially toxic elements (PTEs) (Pb, Cr, and Hg) considered by REACH and RoHS regulations are in Figure 3 and in Appendix A, Table A1. Notably, Sb and Cd were not detected in any item. Indeed, according to the literature, the Sb content was lower in waste mobile phones than in any other WEEE category [44]. Hg was found only in one item (the frame of a feature phone), with a concentration of 0.0017 mg/kg. Cl and Br were the most prominent elements detected, respectively, in 67% of the items, with concentrations between 0.84 mg/kg and 2020 mg/kg and 26% of the items with concentrations ranging between 0.84 mg/kg and 40,700 mg/kg. These findings align with results reported in other studies, where PTEs were found in low concentrations, e.g., between 5 and 340 mg/kg of Pb and 4.6 and 1005 mg/kg for Cd [45], while Br was found in 42% of the samples with concentration ranging between 1.8 and 171,000 mg/kg [46].

**Figure 4:**
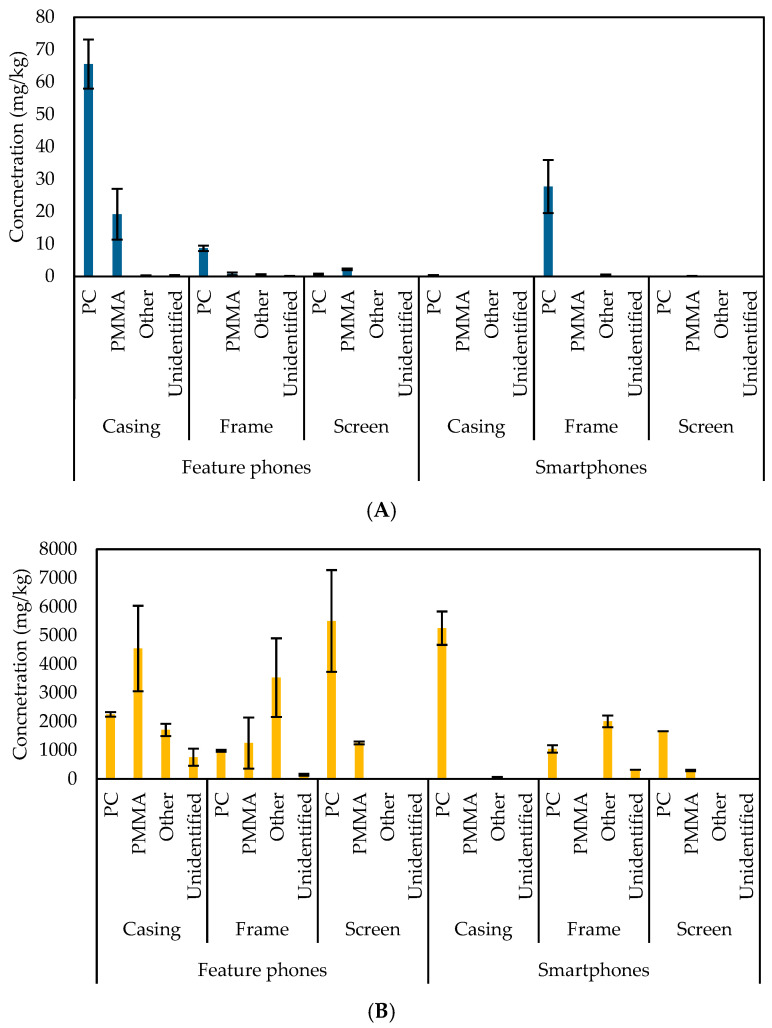

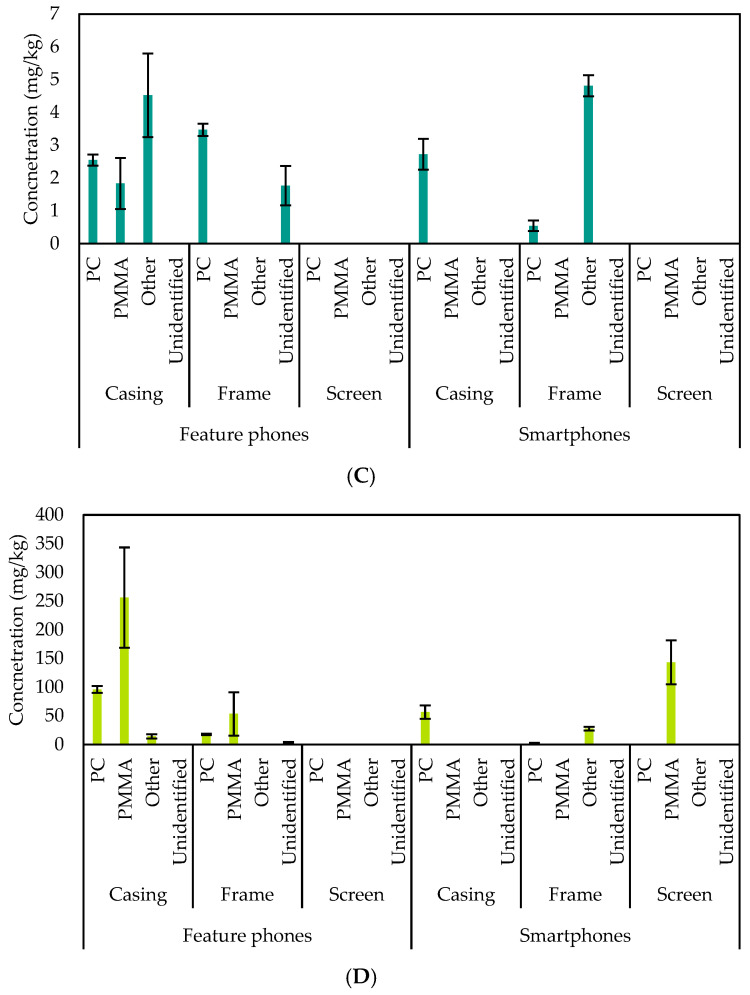
Distribution of the concentration of (**A**) Br, (**B**) Cl, (**C**) Pb, and (**D**) Cr in casings, frames, and screens and in the identified polymers in the plastic components of EoL cell phones considered in this study.

The distribution of the concentration of flame retardants (Br, Cl) and PTEs (Pb, Cr) in the casings, frames, and screens of feature phones and smartphones and in the identified polymers is shown in Figure 4. A significant variation among the concentrations of these elements was observed, consistently with the literature [28], with distinct differences observed between cell phone types and polymers used. This disparity may be attributed to the differences in manufacturing standards, material composition, and performance requirements of feature phones and smartphones.

In feature phones, Br concentrations are low across all components, with the highest value in the casings (65.53 ± 7.55 mg/kg). In contrast, smartphones exhibit the highest concentration of bromine in the frames (27.76 ± 8.19 mg/kg). This suggests a more extensive use of bromine-based flame retardants in smartphone screens, which necessitate enhanced fire safety standards [47], whereas Cl concentrations were higher compared to Br (5504.63 ± 1772.64 mg/kg in feature phones’ casings and 5253.65 ± 582.21 mg/kg in smartphones’ casings). These results indicate the presence of Cl-based additives [47], which are used to enhance structural integrity [48] and improve safety.

Feature phones and smartphones’ plastic screens did not contain Pb. The higher concentration of Pb found in feature phones’ casings (4.52 ± 1.28 mg/kg) and smartphones’ frames (4.8 ± 0.32 mg/kg) could be attributed to the increased complexity and performance of modern devices, which may require materials with a more substantial Pb content to meet the required specifications [45]. Cr concentrations were generally low across both device types (256.09 ± 5.94 mg/kg in feature phones’ casings and 143.22 ± 38.27 mg/kg in smartphones’ screens).

### 3.4. Statistical Analysis

Descriptive statistics, including the number of observations, minimum and maximum values, quartiles, mean, variance, and standard deviation, of the relevant data have been calculated for the entire dataset as well as for relevant subcategories, including samples category (feature phones and smartphones), components (casing, frame, and screen), and polymer fractions (PC, PMMA, and other polymers) (see Appendix A, Table A2, Table A3, Table A4, Table A5, Table A6, Table A7, Table A8, Table A9 and Table A10).

Moreover, Pearson’s correlation coefficients were calculated to evaluate potential relationships between the concentrations of flame retardants and potentially toxic elements in the plastic components of feature phones and smartphones, as well as to investigate possible trends associated with the manufacturing year [34] (see Table 3).

Overall, the analysis revealed low correlation values across all variables, indicating the absence of strong linear associations among the considered factors. The correlation between the year of manufacture and elemental content was consistently weak. The most notable result was a negative correlation of −19% between Br content and the manufacturing year, suggesting a slight decrease in the use of brominated compounds over time. This trend may be attributed to evolving regulatory frameworks such as the RoHS Directive, shifts in manufacturing practices, or increasing reliance on alternative flame-retardant technologies. For other elements (Sb, Cl, Pb, Cr, and Hg) the correlation coefficients with the year of manufacturing were minimal, ranging from −2% to +9%. These negligible values suggest that their presence in plastic components has remained relatively stable over the years or has varied in a non-systematic manner that is not relevant for linear correlation.

Similarly, the inter-element correlations were very low, indicating that the presence and concentration of each element do not predict those of the other ones. The highest observed correlation was between Sb and Pb (16%), which may point to occasional co-use in specific plastic formulations, potentially for synergistic flame-retardant properties or as residuals from manufacturing processes. Notably, Cl content was entirely uncorrelated with both Pb and Cr (0%), suggesting that these additives serve unrelated functions within the plastic components. All other correlations fell within the range of −5% and +5%, reinforcing the interpretation that different additives are incorporated independently and likely fulfill specific purposes that do not systematically overlap.

These findings have several implications. The lack of significant correlation among element concentrations indicates a highly heterogeneous composition of plastic components in mobile phones. This heterogeneity may result from diverse sourcing of polymers, varied design specifications, and differing degrees of regulatory compliance across manufacturers and time periods. Furthermore, the weak correlation with year of manufacture, particularly for Br, may nonetheless provide a tentative indication of progressive substitution of certain legacy additives, although broader datasets and further temporal analysis would be needed to confirm this trend.

## 4. Discussion

The characterization of the inventoried items identified PC and PMMA as the most prevalent polymers; both are thermoplastic materials generally more suitable for recycling compared to thermosetting plastics [49]. However, mixed polymers were also detected in the same items; therefore, a separation of the plastic components into distinct categories by sorting casings, frames, and screens could support recycling operations. Consequently, the use of advanced sorting technologies, such as FTIR [33,35] or hyperspectral imaging [50], is recommended. While FTIR spectroscopy was employed in this study to identify the polymers in plastic components, its applicability extends beyond laboratory-scale characterization. Indeed, in full-scale recycling facilities, FTIR holds considerable potential when integrated with automated sorting systems, due to its non-destructive nature, ease of use, and cost-effectiveness which make it particularly suitable for enhancing the accuracy and efficiency of polymer identification and separation in WEEE plastic streams [51]. FTIR offers a cost-effective solution both for identifying polymer types [12] and detecting harmful additives, such as brominated flame retardants (BFRs) [52,53], which are commonly found in WEEE plastics.

These results highlight the significant prevalence in the plastic components of EoL cell phones of PC and PMMA, as these materials offer exceptional performance in terms of mechanical strength and durability [33,54]. The greater presence of PC in smartphones (59%) compared to feature phones (56%) may suggest that PC’s properties, such as its toughness and resistance to impact, are increasingly valued in the more advanced, larger plastic components that are typical of smartphones, whereas the lower percentage of PMMA in smartphones (15%) compared to feature phones (30%) could indicate a shift towards alternative materials, such as borosilicate glass, to enhance the technical and aesthetic performance of the liquid crystal displays in smartphones [55]. Additionally, the presence of “other polymers” in both categories suggests the use of specific blends or polymers for functional requirements, such as the integration of ABS/PC blends or polyamide for enhanced strength and heat resistance in smartphone frames. Eventually, the diversity of polymers used in frames and casings points to a trend of material customization, driven by the increasing complexity of devices and the demand for enhanced design, durability, and performance.

Although none of the EU regulatory limits for hazardous substances were exceeded, the chlorine content in the samples may raise concerns. Cl reacts with recycling equipment, leading to corrosion, especially during processes like incineration and gasification, which can result in increased maintenance costs and reduced equipment lifespan [56]. Furthermore, the incineration of Cl-containing plastics generates harmful toxic substances, including dioxins and hydrochloric acid, which pose significant environmental and health risks [57]. These toxic emissions hinder the recycling process and require additional treatments to mitigate their impact. To address these challenges, various dechlorination methods have been developed. Techniques such as the melt process with coal tar and iron oxide, the low-temperature critical aqueous ammonia (LCA) process [58], and extraction to form solid Cl salts [59] have proven effective in removing chlorine from plastics, thereby improving recycling outcomes. These technologies not only enable more efficient recycling but also minimize the release of toxic substances, thereby enhancing material recovery. Overall, while Cl in e-waste plastics poses significant barriers to recycling, advancements in dechlorination methods and recycling technologies offer promising solutions for improving both the safety and efficiency of the recycling process [60].

Moreover, while the concentrations of Cl and Pb do not exhibit significant variation across different polymers and components, Br is notably more prevalent in feature phones than in smartphones. In both cell phone types, Pb was prevalent in the PC samples, specifically in the casings of feature phones and the frames of smartphones. A previous study instead identified a positive correlation between the content of Br and the presence of unidentified polymers [61].

Overall, the concentrations of hazardous substances across all components and sample sets were consistently below the established EU regulatory limits. Even in instances where specific samples may exceed the concentration limit for individual components, the weighted average concentrations for the entire sample set, including both feature phones and smartphones, as well as for each individual component, considering casings, frames, and screens, and polymer type, remained well within the prescribed thresholds. This indicates full compliance with the EU legislation regulating recycling standards at all levels, whether targeting individual polymers, specific components, or mixed samples, ensuring that the recycling process adheres to the required safety and environmental guidelines.

## 5. Conclusions

This study provides practical insights into the recyclability potential of plastic components from EoL mobile phones, which represent a significant portion of the WEEE stream. The 275 plastic components of 100 EoL mobile phones (comprising feature phones and smartphones) were analyzed for their polymer composition and hazardous substance content. The most prevalent polymers identified were PC and PMMA, which are both thermoplastic polymers generally suitable for recycling. The concentration of hazardous substances, including flame retardants (Br, Cl, Sb) and potentially toxic elements (PB, Cd, Cr and Hg), was also quantified and compared with the EU regulatory limits set by the RoHS and REACH directives. The results of the characterization of the plastic components showed that, despite the presence of hazardous substances such as Br and Cl in individual items, all concentrations were found to be below the EU regulatory limits for recyclability. This suggests that the plastic components from EoL mobile phones meet the required standards for safe recycling. The variation in hazardous substance concentrations between feature phones and smartphones suggests that tailored recycling strategies may be necessary for different device categories. Moreover, the presence of Cl requires advanced recycling technologies, such as dechlorination methods and specialized recycling processes, to improve the safety of the recycling process.

The findings of this study carry important implications for the recycling and disposal of waste mobile phones. Overall, this study emphasizes the importance of addressing the challenges associated with WEEE plastic recycling, including the need for improved sorting technologies, such as FTIR or hyperspectral imaging, and the development of more efficient and environmentally friendly recycling processes. FTIR is a non-destructive and cost-effective method that can be integrated into automated sorting systems to improve the recycling efficiency of WEEE plastic components. Future efforts should focus on reducing the use of hazardous substances in mobile phones and improving the safety and efficiency of recycling practices, ensuring that the whole environmental impact of WEEE is minimized.

## Figures and Tables

**Table 1:** Regulation limits set by REACH and RoHS for flame retardants and potentially toxic elements considered to assess the recyclability potential of plastic components from EoL mobile phones.

Substance of Concerns	Element	Threshold Limit	Ref.
Flame retardant	Br	1000 ppm	[37]
	Cl	1000 ppm	[37]
	Sb	8300 ppm	[38]
Potentially toxic element	Cd	0.01%wt.	[14]
	Cr	0.1%wt.	[14]
	Hg	0.1%wt.	[14]
	Pb	0.1%wt.	[14]

**Table 2:** Mass balance of the plastic components considered in this study.

	Feature Phones			Smartphones		
	Casing	Frame	Screen	Casing	Frame	Screen
Mass of components	0.67 kg	1.10 kg	0.82 kg	0.32 kg	0.34 kg	0.12 kg
Mass of samples category	2.58 kg			0.79 kg		
Mass of sample set	3.37 kg					

**Table 3:** Pearson’s correlation among Br, Sb, Cl, Cr, Pb, and Hg in mobile phone (feature phones and smartphones) plastic components.

	Br	Sb	Cl	Pb	Cr	Hg
Years	−19%	2%	6%	3%	9%	−1%
Br	-	−3%	−1%	−1%	−3%	−1%
Sb		-	3%	16%	2%	−2%
Cl			-	0%	0%	−3%
Pb				-	−1%	−2%
Cr					-	−2%

## Data Availability

Data can be made available upon request.

## Appendix A

**Table A1:** Inventory of samples and characterization results: mass (g), identified polymers, and concentration (mg/kg) of Br, Sb, Cl, Pb, Cr, Cd, and Hg.

Model	Component	Sample Set	Mass (g)	Polymer	Br (mg/kg)	Sb (mg/kg)	Cl (mg/kg)	Pb (mg/kg)	Cr (mg/kg)	Cd (mg/kg)	Hg (mg/kg)
Alcatel One touch 556	Casing	Feature phone	10.45	Other polymers	0.00	0.00	1240.00	28.00	0.00	0.00	0.00
Apple iPhone 4	Casing	Feature phone	8.82	Polycarbonate	0.00	0.00	0.00	0.00	0.00	0.00	0.00
BlackBerry 9360	Casing	Feature phone	8.75	Other polymers	0.00	0.00	0.00	0.00	0.00	0.00	0.00
Blackberry 98310	Casing	Feature phone	10.42	Poly(methyl methacrylate)	0.00	0.00	2030.00	0.00	218.00	0.00	0.00
Bosh Dual Com738	Casing	Feature phone	20.56	Polycarbonate	0.48	0.00	1100.00	0.00	0.00	0.00	0.00
BrionVega 7010	Casing	Feature phone	15.77	Polycarbonate	1.10	0.00	8470.00	0.00	0.00	0.00	0.00
Brondi Gladietor 3	Casing	Feature phone	9.78	Polycarbonate	1.61	0.00	3110.00	0.00	0.00	0.00	0.00
Cross Call	Casing	Feature phone	3.27	Polycarbonate	0.00	0.00	996.00	0.00	608.00	0.00	0.00
Ericsson 5868	Casing	Feature phone	12.89	Polycarbonate	0.00	0.00	17,600.00	0.00	149.00	0.00	0.00
Ericsson a1018	Casing	Feature phone	4.89	Polycarbonate	0.00	0.00	0.00	0.00	0.00	0.00	0.00
Ericsson s868	Casing	Feature phone	11.11	Poly(methyl methacrylate)	0.00	0.00	2280.00	0.00	0.00	0.00	0.00
Ericsson s868	Casing	Feature phone	25.63	Polycarbonate	0.90	0.00	0.00	0.00	0.00	0.00	0.00
Htc desire 316	Casing	Feature phone	12.53	Poly(methyl methacrylate)	0.00	0.00	0.00	0.00	0.00	0.00	0.00
LG L3431	Casing	Feature phone	24.69	Polycarbonate	16.70	0.00	2460.00	0.00	164.00	0.00	0.00
LG u8110	Casing	Feature phone	7.26	Polycarbonate	0.00	0.00	0.00	0.00	0.00	0.00	0.00
Nokia 5230	Casing	Feature phone	13.03	Polycarbonate	0.00	0.00	874.00	0.00	0.00	0.00	0.00
Motorola	Casing	Feature phone	14.29	Poly(methyl methacrylate)	0.71	0.00	15,800.00	8.00	915.00	0.00	0.00
Motorola c350	Casing	Feature phone	9.08	Polycarbonate	0.00	0.00	1110.00	0.00	0.00	0.00	0.00
Motorola cd930	Casing	Feature phone	4.14	Polycarbonate	0.00	0.00	0.00	0.00	0.00	0.00	0.00
Motorola D520	Casing	Feature phone	10.50	Polycarbonate	0.20	0.00	1090.00	0.00	149.00	0.00	0.00
Motorola Micro Tack	Casing	Feature phone	10.46	Polycarbonate	0.00	0.00	3660.00	6.00	0.00	0.00	0.00
Motorola Star Tack	Casing	Feature phone	3.76	Polycarbonate	0.00	0.00	0.00	0.00	0.00	0.00	0.00
Motorola Star Tack	Casing	Feature phone	5.29	Other polymers	0.00	0.00	0.00	0.00	0.00	0.00	0.00
Motorola Startack 8700	Casing	Feature phone	5.65	Polycarbonate	353.00	0.00	4130.00	15.00	0.00	0.00	0.00
Motorola Talkabout	Casing	Feature phone	10.54	Polycarbonate	0.00	0.00	1060.00	0.00	0.00	0.00	0.00
NGM Dinamic	Casing	Feature phone	11.14	Polycarbonate	0.00	0.00	0.00	0.00	0.00	0.00	0.00
Nokia 1110	Casing	Feature phone	11.04	Polycarbonate	0.00	0.00	2550.00	52.00	65.00	0.00	0.00
Nokia 1110i	Casing	Feature phone	3.34	Polycarbonate	0.00	0.00	1140.00	0.00	426.00	0.00	0.00
Nokia 1110i	Casing	Feature phone	4.02	Polycarbonate	0.00	0.00	805.00	0.00	41.00	0.00	0.00
Nokia 1200	Casing	Feature phone	5.17	Polycarbonate	0.00	0.00	1700.00	0.00	0.00	0.00	0.00
Nokia 1209	Casing	Feature phone	0.88	Polycarbonate	0.00	0.00	1230.00	0.00	0.00	0.00	0.00
Nokia 2100	Casing	Feature phone	12.68	Polycarbonate	2.59	0.00	1800.00	0.00	0.00	0.00	0.00
Nokia 2330	Casing	Feature phone	13.63	Polycarbonate	29.80	0.00	4750.00	14.00	27.00	0.00	0.00
Nokia 2600	Casing	Feature phone	6.20	Polycarbonate	0.00	0.00	0.00	0.00	0.00	0.00	0.00
Nokia 2630	Casing	Feature phone	2.97	Polycarbonate	0.00	0.00	0.00	0.00	0.00	0.00	0.00
Nokia 2630	Casing	Feature phone	5.65	Polycarbonate	3.82	0.00	1050.00	0.00	0.00	0.00	0.00
Nokia 2650	Casing	Feature phone	9.14	Polycarbonate	2.86	0.00	1180.00	0.00	11.00	0.00	0.00
Nokia 2760	Casing	Feature phone	16.79	Polycarbonate	0.49	0.00	206.00	0.00	0.00	0.00	0.00
Nokia 301	Casing	Feature phone	5.93	Polycarbonate	0.00	0.00	0.00	0.00	0.00	0.00	0.00
Nokia 3210	Casing	Feature phone	8.93	Polycarbonate	0.00	0.00	1890.00	26.00	28.00	0.00	0.00
Nokia 3220	Casing	Feature phone	10.94	Polycarbonate	0.00	0.00	1180.00	0.00	0.00	0.00	0.00
Nokia 3220	Casing	Feature phone	12.83	Polycarbonate	1.89	0.00	7090.00	5.00	0.00	0.00	0.00
Nokia 3310	Casing	Feature phone	10.27	Polycarbonate	0.46	0.00	849.00	0.00	467.00	0.00	0.00
Nokia 3310	Casing	Feature phone	5.86	Polycarbonate	0.00	0.00	0.00	0.00	0.00	0.00	0.00
Nokia 3510	Casing	Feature phone	8.87	Polycarbonate	0.00	0.00	0.00	0.00	0.00	0.00	0.00
Nokia 5230	Casing	Feature phone	0.70	Polycarbonate	0.00	0.00	2080.00	15.00	0.00	0.00	0.00
Nokia 5530	Casing	Feature phone	4.68	Other polymers	1.60	0.00	8740.00	0.00	0.00	0.00	0.00
Nokia 6021	Casing	Feature phone	3.42	Polycarbonate	5.26	0.00	426.00	0.00	0.00	0.00	0.00
Nokia 6600	Casing	Feature phone	5.17	Polycarbonate	2.48	0.00	621.00	0.00	0.00	0.00	0.00
Nokia 8210	Casing	Feature phone	5.10	Polycarbonate	3.69	0.00	4390.00	7.00	33.00	0.00	0.00
Nokia C1-02	Casing	Feature phone	0.91	Polycarbonate	5.29	0.00	4650.00	7.00	35.00	0.00	0.00
Nokia c202	Casing	Feature phone	3.07	Polycarbonate	0.57	0.00	654.00	0.00	0.00	0.00	0.00
Nokia C503	Casing	Feature phone	12.29	Polycarbonate	0.00	0.00	0.00	0.00	0.00	0.00	0.00
Nokia C504	Casing	Feature phone	3.97	Polycarbonate	0.00	0.00	0.00	0.00	0.00	0.00	0.00
Nokia E61i	Casing	Feature phone	3.20	Polycarbonate	0.00	0.00	738.00	0.00	0.00	0.00	0.00
Nokia E61i	Casing	Feature phone	7.43	Polycarbonate	2.83	0.00	11,300.00	0.00	0.00	0.00	0.00
Nokia E61i	Casing	Feature phone	28.72	Polycarbonate	1.57	0.00	1230.00	1.00	470.00	0.00	0.00
Nokia E61i	Casing	Feature phone	14.79	Other polymers	0.95	0.00	1750.00	0.00	0.00	0.00	0.00
Nokia N70	Casing	Feature phone	7.69	Polycarbonate	0.00	0.00	1980.00	0.00	0.00	0.00	0.00
Nokia N70	Casing	Feature phone	5.30	Other polymers	0.00	0.00	2990.00	0.00	0.00	0.00	0.00
Nokia N70	Casing	Feature phone	3.19	Poly(methyl methacrylate)	0.00	0.00	0.00	0.00	0.00	0.00	0.00
Nokia N70	Casing	Feature phone	8.38	Poly(methyl methacrylate)	137.00	0.00	0.00	0.00	0.00	0.00	0.00
Nokia N73	Casing	Feature phone	6.06	Other polymers	0.00	0.00	0.00	3.00	144.00	0.00	0.00
Nokia N97	Casing	Feature phone	4.13	Polycarbonate	0.00	0.00	0.00	0.00	0.00	0.00	0.00
Onda n4020	Casing	Feature phone	8.10	Polycarbonate	17.20	0.00	1380.00	0.00	0.00	0.00	0.00
Panasonic G450	Casing	Feature phone	7.13	Polycarbonate	0.00	0.00	532.00	0.00	0.00	0.00	0.00
Panasonic GD87	Casing	Feature phone	24.25	Other polymers	0.00	0.00	1670.00	2.00	11.00	0.00	0.00
Philips Fisio 120	Casing	Feature phone	6.01	Polycarbonate	0.00	0.00	0.00	0.00	0.00	0.00	0.00
Philips Genie 2000	Casing	Feature phone	14.30	Polycarbonate	2020.00	0.00	822.00	0.00	0.00	0.00	0.00
Sagem MyX3-2	Casing	Feature phone	8.88	Polycarbonate	121.00	0.00	2120.00	0.00	0.00	0.00	0.00
Samsung Gt e1200	Casing	Feature phone	9.34	Polycarbonate	0.00	0.00	6170.00	0.00	0.00	0.00	0.00
Nokia N70	Frame	Feature phone	19.98	Polycarbonate	0.00	0.00	124.00	0.00	0.00	0.00	0.00
Nokia E61i	Frame	Feature phone	10.72	Polycarbonate	0.00	0.00	130.00	0.00	0.00	0.00	0.00
Nokia 3210	Frame	Feature phone	14.06	Polycarbonate	0.00	0.00	176.00	0.00	0.00	0.00	0.00
Sagem sg 850	Frame	Feature phone	18.42	Polycarbonate	0.00	0.00	194.00	0.00	0.00	0.00	0.00
Nokia N70	Frame	Feature phone	13.62	Other polymers	0.00	0.00	198.00	0.00	0.00	0.00	0.00
Htc desire 316	Frame	Feature phone	16.91	Other polymers	0.00	0.00	202.00	0.00	0.00	0.00	0.00
BrionVega 7010	Frame	Feature phone	13.38	Polycarbonate	0.00	0.00	202.00	0.00	0.00	0.00	0.00
Nokia 3220	Frame	Feature phone	20.19	Polycarbonate	0.00	0.00	229.00	0.00	0.00	0.00	0.00
Nokia 2600	Frame	Feature phone	11.97	Polycarbonate	0.00	0.00	238.00	0.00	0.00	0.00	0.00
Nokia E61i	Frame	Feature phone	19.18	Polycarbonate	0.00	0.00	256.00	0.00	0.00	0.00	0.00
Motorola Talkabout	Frame	Feature phone	13.95	Polycarbonate	0.00	0.00	304.00	0.00	0.00	0.00	0.00
Ericsson 5868	Frame	Feature phone	18.70	Other polymers	0.00	0.00	318.00	0.00	0.00	0.00	17.00
Nokia 5230	Frame	Feature phone	11.29	Polycarbonate	0.00	0.00	338.00	0.00	0.00	0.00	0.00
Onda n4020	Frame	Feature phone	11.49	Polycarbonate	0.00	0.00	381.00	0.00	0.00	0.00	0.00
Motorola Startack 8700	Frame	Feature phone	13.17	Polycarbonate	0.00	0.00	404.00	0.00	0.00	0.00	0.00
Nokia 3310	Frame	Feature phone	11.82	Polycarbonate	0.00	0.00	497.00	0.00	0.00	0.00	0.00
Nokia 8210	Frame	Feature phone	4.56	Other polymers	0.00	0.00	0.00	0.00	0.00	0.00	0.00
Nokia E61i	Frame	Feature phone	15.95	Other polymers	0.00	0.00	0.00	0.00	0.00	0.00	0.00
Blackberry 98310	Frame	Feature phone	12.33	Poly(methyl methacrylate)	0.00	0.00	0.00	0.00	0.00	0.00	0.00
Alcatel One touch 556	Frame	Feature phone	16.09	Polycarbonate	0.00	0.00	0.00	0.00	0.00	0.00	0.00
Ericsson s868	Frame	Feature phone	10.82	Polycarbonate	0.00	0.00	0.00	0.00	0.00	0.00	0.00
Motorola c350	Frame	Feature phone	11.77	Polycarbonate	0.00	0.00	0.00	0.00	0.00	0.00	0.00
Motorola D520	Frame	Feature phone	14.79	Polycarbonate	0.00	0.00	0.00	0.00	0.00	0.00	0.00
Motorola Micro Tack	Frame	Feature phone	10.75	Polycarbonate	0.00	0.00	0.00	0.00	0.00	0.00	0.00
Nokia 1110i	Frame	Feature phone	10.22	Polycarbonate	0.00	0.00	0.00	0.00	0.00	0.00	0.00
Nokia 2100	Frame	Feature phone	19.59	Polycarbonate	0.00	0.00	0.00	0.00	0.00	0.00	0.00
Nokia 2630	Frame	Feature phone	12.13	Polycarbonate	0.00	0.00	0.00	0.00	0.00	0.00	0.00
Nokia 2760	Frame	Feature phone	18.59	Polycarbonate	0.00	0.00	0.00	0.00	0.00	0.00	0.00
Nokia 3220	Frame	Feature phone	13.93	Polycarbonate	0.00	0.00	0.00	0.00	0.00	0.00	0.00
Nokia 3310	Frame	Feature phone	10.39	Polycarbonate	0.00	0.00	0.00	0.00	0.00	0.00	0.00
Nokia 3510	Frame	Feature phone	11.63	Polycarbonate	0.00	0.00	0.00	0.00	0.00	0.00	0.00
Nokia 5530	Frame	Feature phone	15.95	Polycarbonate	0.00	0.00	0.00	0.00	0.00	0.00	0.00
Nokia 6021	Frame	Feature phone	10.71	Polycarbonate	0.00	0.00	0.00	0.00	0.00	0.00	0.00
Nokia 6600	Frame	Feature phone	9.96	Polycarbonate	0.00	0.00	0.00	0.00	0.00	0.00	0.00
Nokia C1-02	Frame	Feature phone	9.15	Polycarbonate	0.00	0.00	0.00	0.00	0.00	0.00	0.00
Nokia c202	Frame	Feature phone	12.11	Polycarbonate	0.00	0.00	0.00	0.00	0.00	0.00	0.00
Nokia N70	Frame	Feature phone	20.22	Polycarbonate	0.00	0.00	0.00	0.00	0.00	0.00	0.00
Panasonic GD87	Frame	Feature phone	14.45	Polycarbonate	0.00	0.00	0.00	0.00	0.00	0.00	0.00
Philips Genie 2000	Frame	Feature phone	30.32	Polycarbonate	0.00	0.00	0.00	0.00	0.00	0.00	0.00
Sagem MyX3-2	Frame	Feature phone	16.76	Polycarbonate	0.00	0.00	0.00	0.00	0.00	0.00	0.00
Samsung Gt e1200	Frame	Feature phone	18.44	Polycarbonate	0.00	0.00	0.00	0.00	0.00	0.00	0.00
Nokia 1110	Frame	Feature phone	22.41	Polycarbonate	0.00	0.00	834.00	0.00	0.00	0.00	0.00
Mnokia 5230	Frame	Feature phone	14.23	Polycarbonate	0.00	0.00	1230.00	0.00	0.00	0.00	0.00
Motorola cd930	Frame	Feature phone	14.88	Polycarbonate	0.00	0.00	1410.00	0.00	0.00	0.00	0.00
Motorola Star Tack	Frame	Feature phone	10.01	Polycarbonate	0.55	0.00	1510.00	0.00	0.00	0.00	0.00
Nokia 1110i	Frame	Feature phone	15.08	Polycarbonate	8.43	0.00	1820.00	0.00	0.00	0.00	0.00
Nokia E61i	Frame	Feature phone	11.82	Polycarbonate	0.00	0.00	2670.00	0.00	0.00	0.00	0.00
Brondi Gladietor 3	Frame	Feature phone	12.54	Other polymers	3.11	0.00	22,200.00	0.00	0.00	0.00	0.00
Nokia N70	Frame	Feature phone	19.39	Polycarbonate	25.50	0.00	650.00	0.00	9.00	0.00	0.00
NGM Dinamic	Frame	Feature phone	13.54	Polycarbonate	27.00	0.00	710.00	0.00	31.00	0.00	0.00
BlackBerry 9360	Frame	Feature phone	13.03	Poly(methyl methacrylate)	1.30	0.00	2440.00	0.00	104.00	0.00	0.00
Nokia 2630	Frame	Feature phone	13.27	Polycarbonate	7.00	0.00	2200.00	0.00	559.00	0.00	0.00
Apple iphone 4	Frame	Feature phone	8.41	Polycarbonate	0.00	0.00	1650.00	2.00	0.00	0.00	0.00
Motorola	Frame	Feature phone	13.77	Polycarbonate	370.00	0.00	354.00	4.00	0.00	0.00	0.00
Nokia 2650	Frame	Feature phone	13.52	Polycarbonate	4.04	0.00	0.00	4.00	0.00	0.00	0.00
Nokia 1209	Frame	Feature phone	11.58	Polycarbonate	2.56	0.00	539.00	6.00	41.00	0.00	0.00
Nokia C503	Frame	Feature phone	14.30	Polycarbonate	0.53	0.00	627.00	6.00	111.00	0.00	0.00
Nokia C504	Frame	Feature phone	19.03	Polycarbonate	0.82	0.00	5380.00	8.00	34.00	0.00	0.00
Philips Fisio 120	Frame	Feature phone	18.70	Polycarbonate	3.17	0.00	4390.00	8.00	39.00	0.00	0.00
Nokia 2330	Frame	Feature phone	21.62	Polycarbonate	31.00	0.00	4470.00	13.00	28.00	0.00	0.00
Nokia 301	Frame	Feature phone	18.62	Polycarbonate	0.79	0.00	5250.00	19.00	50.00	0.00	0.00
Nokia 1200	Frame	Feature phone	18.77	Polycarbonate	5.01	0.00	6070.00	20.00	39.00	0.00	0.00
Panasonic G450	Frame	Feature phone	13.95	Polycarbonate	0.00	0.00	3620.00	22.00	48.00	0.00	0.00
Nokia N97	Frame	Feature phone	10.82	Polycarbonate	6.69	0.00	0.00	90.00	33.00	0.00	0.00
NGM Dinamic	Screen	Feature phone	10.16	Poly(methyl methacrylate)	0.00	0.00	0.84	0.00	0.00	0.00	0.00
Panasonic G450	Screen	Feature phone	10.46	Poly(methyl methacrylate)	0.00	0.00	112.00	0.00	0.00	0.00	0.00
Nokia E61i	Screen	Feature phone	14.38	Polycarbonate	1.85	0.00	164.00	0.00	0.00	0.00	0.00
Nokia N73	Screen	Feature phone	14.97	Poly(methyl methacrylate)	0.00	0.00	172.00	0.00	0.00	0.00	0.00
Bosh Dual Com738	Screen	Feature phone	16.37	Poly(methyl methacrylate)	0.00	0.00	182.00	0.00	0.00	0.00	0.00
Nokia C1-02	Screen	Feature phone	6.86	Poly(methyl methacrylate)	0.00	0.00	186.00	0.00	0.00	0.00	0.00
BrionVega 7010	Screen	Feature phone	10.03	Poly(methyl methacrylate)	0.00	0.00	192.00	0.00	0.00	0.00	0.00
Nokia 1200	Screen	Feature phone	14.08	Poly(methyl methacrylate)	0.00	0.00	193.00	0.00	0.00	0.00	0.00
Nokia 5530	Screen	Feature phone	11.96	Poly(methyl methacrylate)	0.00	0.00	196.00	0.00	0.00	0.00	0.00
Nokia 301	Screen	Feature phone	13.96	Poly(methyl methacrylate)	0.00	0.00	202.00	0.00	0.00	0.00	0.00
Nokia 3310	Screen	Feature phone	8.86	Poly(methyl methacrylate)	0.00	0.00	205.00	0.00	0.00	0.00	0.00
Motorola Star Tack	Screen	Feature phone	12.04	Poly(methyl methacrylate)	0.00	0.00	211.00	0.00	0.00	0.00	0.00
Nokia N70	Screen	Feature phone	14.55	Poly(methyl methacrylate)	0.00	0.00	216.00	0.00	0.00	0.00	0.00
Philips Fisio 120	Screen	Feature phone	14.03	Poly(methyl methacrylate)	0.00	0.00	231.00	0.00	0.00	0.00	0.00
Motorola	Screen	Feature phone	10.33	Poly(methyl methacrylate)	0.00	0.00	243.00	0.00	0.00	0.00	0.00
Onda n4020	Screen	Feature phone	8.62	Poly(methyl methacrylate)	0.00	0.00	252.00	0.00	0.00	0.00	0.00
Nokia 3220	Screen	Feature phone	10.45	Poly(methyl methacrylate)	0.00	0.00	268.00	0.00	0.00	0.00	0.00
BlackBerry 9360	Screen	Feature phone	9.77	Poly(methyl methacrylate)	0.00	0.00	297.00	0.00	0.00	0.00	0.00
Nokia N70	Screen	Feature phone	14.99	Poly(methyl methacrylate)	0.00	0.00	328.00	0.00	0.00	0.00	0.00
Motorola Talkabout	Screen	Feature phone	10.46	Poly(methyl methacrylate)	0.00	0.00	342.00	0.00	0.00	0.00	0.00
CrossCall	Screen	Feature phone	14.37	Poly(methyl methacrylate)	0.00	0.00	368.00	0.00	0.00	0.00	0.00
Nokia 3210	Screen	Feature phone	10.54	Poly(methyl methacrylate)	0.00	0.00	422.00	0.00	0.00	0.00	0.00
Ericsson s868	Screen	Feature phone	15.07	Polycarbonate	1.96	0.00	474.00	0.00	0.00	0.00	0.00
Nokia 2630	Screen	Feature phone	9.95	Poly(methyl methacrylate)	0.00	0.00	482.00	0.00	0.00	0.00	0.00
Alcatel One touch 556	Screen	Feature phone	12.06	Polycarbonate	0.00	0.00	520.00	0.00	0.00	0.00	0.00
Motorola c350	Screen	Feature phone	8.83	Poly(methyl methacrylate)	0.00	0.00	558.00	0.00	0.00	0.00	0.00
Nokia 2630	Screen	Feature phone	9.10	Poly(methyl methacrylate)	0.00	0.00	563.00	0.00	0.00	0.00	0.00
Sagem sg 850	Screen	Feature phone	13.82	Poly(methyl methacrylate)	110.00	0.00	573.00	0.00	0.00	0.00	0.00
LG u8110	Screen	Feature phone	12.99	Poly(methyl methacrylate)	0.00	0.00	595.00	0.00	0.00	0.00	0.00
Motorola cd930	Screen	Feature phone	11.16	Poly(methyl methacrylate)	0.00	0.00	620.00	0.00	0.00	0.00	0.00
Nokia 2330	Screen	Feature phone	16.22	Poly(methyl methacrylate)	0.00	0.00	663.00	0.00	0.00	0.00	0.00
Blackberry 98310	Screen	Feature phone	9.25	Poly(methyl methacrylate)	0.00	0.00	666.00	0.00	0.00	0.00	0.00
Htc desire 316	Screen	Feature phone	12.68	Polycarbonate	0.00	0.00	666.00	0.00	0.00	0.00	0.00
Brondi Gladietor 3	Screen	Feature phone	9.41	Poly(methyl methacrylate)	0.00	0.00	746.00	0.00	0.00	0.00	0.00
Nokia N97	Screen	Feature phone	8.12	Polycarbonate	0.00	0.00	769.00	0.00	0.00	0.00	0.00
Ericsson s868	Screen	Feature phone	8.12	Poly(methyl methacrylate)	0.00	0.00	0.00	0.00	0.00	0.00	0.00
Mnokia 5230	Screen	Feature phone	10.67	Poly(methyl methacrylate)	0.00	0.00	0.00	0.00	0.00	0.00	0.00
Nokia 1110i	Screen	Feature phone	11.31	Poly(methyl methacrylate)	0.00	0.00	0.00	0.00	0.00	0.00	0.00
Nokia 1209	Screen	Feature phone	8.69	Poly(methyl methacrylate)	0.00	0.00	0.00	0.00	0.00	0.00	0.00
Nokia 2650	Screen	Feature phone	10.14	Poly(methyl methacrylate)	0.00	0.00	0.00	0.00	0.00	0.00	0.00
Nokia 3220	Screen	Feature phone	15.15	Poly(methyl methacrylate)	0.00	0.00	0.00	0.00	0.00	0.00	0.00
Nokia 3510	Screen	Feature phone	8.72	Poly(methyl methacrylate)	0.00	0.00	0.00	0.00	0.00	0.00	0.00
Nokia 5230	Screen	Feature phone	8.46	Poly(methyl methacrylate)	0.00	0.00	0.00	0.00	0.00	0.00	0.00
Nokia 6600	Screen	Feature phone	7.47	Poly(methyl methacrylate)	0.00	0.00	0.00	0.00	0.00	0.00	0.00
Nokia 8210	Screen	Feature phone	3.32	Poly(methyl methacrylate)	0.00	0.00	0.00	0.00	0.00	0.00	0.00
Nokia c202	Screen	Feature phone	5.65	Poly(methyl methacrylate)	0.00	0.00	0.00	0.00	0.00	0.00	0.00
Nokia N70	Screen	Feature phone	15.17	Poly(methyl methacrylate)	0.00	0.00	0.00	0.00	0.00	0.00	0.00
Nokia N70	Screen	Feature phone	10.22	Poly(methyl methacrylate)	0.00	0.00	0.00	0.00	0.00	0.00	0.00
Nokia N73	Screen	Feature phone	9.73	Poly(methyl methacrylate)	0.00	0.00	0.00	0.00	0.00	0.00	0.00
Panasonic GD87	Screen	Feature phone	10.84	Poly(methyl methacrylate)	0.00	0.00	0.00	0.00	0.00	0.00	0.00
Philips Genie 2000	Screen	Feature phone	22.74	Poly(methyl methacrylate)	0.00	0.00	0.00	0.00	0.00	0.00	0.00
Samsung Gt e1200	Screen	Feature phone	9.83	Poly(methyl methacrylate)	0.00	0.00	0.00	0.00	0.00	0.00	0.00
Nokia 2760	Screen	Feature phone	13.95	Polycarbonate	0.00	0.00	0.00	0.00	0.00	0.00	0.00
Apple iphone 4	Screen	Feature phone	6.31	Poly(methyl methacrylate)	0.00	0.00	936.00	0.00	0.00	0.00	0.00
Motorola Startack 8700	Screen	Feature phone	9.88	Poly(methyl methacrylate)	0.00	0.00	972.00	0.00	0.00	0.00	0.00
Ericsson a1018	Screen	Feature phone	12.26	Poly(methyl methacrylate)	0.00	0.00	1050.00	0.00	0.00	0.00	0.00
LG L3431	Screen	Feature phone	15.03	Polycarbonate	1.78	0.00	1070.00	0.00	0.00	0.00	0.00
Motorola D520	Screen	Feature phone	11.09	Poly(methyl methacrylate)	0.00	0.00	1450.00	0.00	0.00	0.00	0.00
Nokia C504	Screen	Feature phone	14.27	Poly(methyl methacrylate)	0.00	0.00	1510.00	0.00	0.00	0.00	0.00
Nokia E61i	Screen	Feature phone	8.86	Poly(methyl methacrylate)	0.00	0.00	1910.00	0.00	0.00	0.00	0.00
Nokia 2600	Screen	Feature phone	8.98	Poly(methyl methacrylate)	0.00	0.00	1950.00	0.00	0.00	0.00	0.00
Ericsson 5868	Screen	Feature phone	14.03	Poly(methyl methacrylate)	0.00	0.00	2370.00	0.00	0.00	0.00	0.00
Nokia E61i	Screen	Feature phone	11.97	Poly(methyl methacrylate)	0.00	0.00	2670.00	0.00	0.00	0.00	0.00
Motorola Star Tack	Screen	Feature phone	7.51	Poly(methyl methacrylate)	0.00	0.00	2690.00	0.00	0.00	0.00	0.00
Nokia C503	Screen	Feature phone	10.73	Poly(methyl methacrylate)	0.00	0.00	3130.00	0.00	0.00	0.00	0.00
Nokia 1110i	Screen	Feature phone	7.66	Poly(methyl methacrylate)	0.00	0.00	3250.00	0.00	0.00	0.00	0.00
Nokia 6021	Screen	Feature phone	8.04	Poly(methyl methacrylate)	0.00	0.00	3480.00	0.00	0.00	0.00	0.00
Nokia 2100	Screen	Feature phone	14.69	Poly(methyl methacrylate)	0.00	0.00	3510.00	0.00	0.00	0.00	0.00
Nokia 3310	Screen	Feature phone	7.79	Poly(methyl methacrylate)	0.00	0.00	3710.00	0.00	0.00	0.00	0.00
Sagem MyX3-2	Screen	Feature phone	12.57	Poly(methyl methacrylate)	0.76	0.00	3830.00	0.00	0.00	0.00	0.00
Nokia E61i	Screen	Feature phone	8.04	Poly(methyl methacrylate)	0.00	0.00	15,400.00	0.00	0.00	0.00	0.00
Motorola Micro Tack	Screen	Feature phone	8.06	Poly(methyl methacrylate)	0.00	0.00	30,300.00	0.00	0.00	0.00	0.00
Nokia 1110	Screen	Feature phone	16.81	Polycarbonate	0.00	0.00	32,600.00	0.00	0.00	0.00	0.00
Samsung GT53650	Casing	Smartphone	12.15	Polycarbonate	1.22	0.00	3700.00	0.00	0.37	0.00	0.00
Samsung GT55230	Casing	Smartphone	15.74	Polycarbonate	0.00	0.00	0.00	0.00	0.00	0.00	0.00
Samsung GTC3500	Casing	Smartphone	13.17	Polycarbonate	0.18	0.00	40,700.00	0.00	0.00	0.00	0.00
Samsung Gti 9300	Casing	Smartphone	8.88	Polycarbonate	1.51	0.00	2660.00	0.00	0.00	0.00	0.00
Samsung GTi9195	Casing	Smartphone	9.22	Other polymers	0.00	0.00	0.00	0.00	0.00	0.00	0.00
Samsung J120 F Galaxy	Casing	Smartphone	5.05	Other polymers	0.00	0.00	0.00	0.00	0.00	0.00	0.00
Samsung sgh e700	Casing	Smartphone	33.84	Polycarbonate	0.00	0.00	1610.00	13.00	99.00	0.00	0.00
Samsung sgh250	Casing	Smartphone	2.61	Polycarbonate	0.00	0.00	0.00	0.00	0.00	0.00	0.00
Samsung sghe530	Casing	Smartphone	1.53	Polycarbonate	0.00	0.00	0.00	0.00	0.00	0.00	0.00
Siemens A35	Casing	Smartphone	14.19	Polycarbonate	0.00	0.00	620.00	0.00	0.00	0.00	0.00
Siemens A50	Casing	Smartphone	7.09	Polycarbonate	0.00	0.00	1900.00	0.00	0.00	0.00	0.00
Siemens c62	Casing	Smartphone	9.88	Polycarbonate	0.54	0.00	35,100.00	0.00	0.00	0.00	0.00
Siemens CFX65	Casing	Smartphone	14.94	Polycarbonate	0.00	0.00	0.00	0.00	0.00	0.00	0.00
Sony CMD Z5	Casing	Smartphone	5.70	Polycarbonate	0.00	0.00	0.00	0.00	0.00	0.00	0.00
Sony ericsson c200	Casing	Smartphone	46.67	Polycarbonate	0.00	0.00	0.00	0.00	0.00	0.00	0.00
Sony ericsson z1010	Casing	Smartphone	0.70	Other polymers	0.00	0.00	994.00	0.00	0.00	0.00	0.00
Wiko_Barry	Casing	Smartphone	29.09	Polycarbonate	0.00	0.00	0.00	0.00	0.00	0.00	0.00
Wuawei Ascend G630	Casing	Smartphone	80.41	Polycarbonate	0.85	0.00	2210.00	5.00	0.00	0.00	0.00
ZTE Blade C2 plus	Casing	Smartphone	12.03	Polycarbonate	0.00	0.00	585.00	0.00	0.00	0.00	0.00
Siemens CFX65	Frame	Smartphone	13.54	Polycarbonate	0.12	0.00	624.00	0.00	0.00	0.00	0.00
Wiko_Barry	Frame	Smartphone	19.95	Polycarbonate	0.29	0.00	667.00	0.00	0.00	0.00	0.00
Samsung GT53650	Frame	Smartphone	21.16	Other polymers	0.00	0.00	0.00	0.00	0.00	0.00	0.00
Siemens A35	Frame	Smartphone	14.24	Polycarbonate	0.00	0.00	0.00	0.00	0.00	0.00	0.00
Siemens A50	Frame	Smartphone	13.81	Polycarbonate	0.00	0.00	0.00	0.00	0.00	0.00	0.00
Siemens s25	Frame	Smartphone	22.93	Polycarbonate	0.00	0.00	875.00	0.00	0.00	0.00	0.00
Siemens c62	Frame	Smartphone	18.25	Polycarbonate	0.00	0.00	1410.00	0.00	0.00	0.00	0.00
Sony D2005	Frame	Smartphone	20.50	Polycarbonate	182.00	0.00	3100.00	0.00	0.00	0.00	0.00
Samsung sgh250	Frame	Smartphone	6.18	Other polymers	0.00	0.00	1340.00	0.00	28.00	0.00	0.00
Samsung Gti9195	Frame	Smartphone	15.73	Other polymers	0.08	0.00	1430.00	1.00	0.00	0.00	0.00
Wuawei Ascend G630	Frame	Smartphone	9.09	Polycarbonate	48.40	0.00	534.00	1.00	37.00	0.00	0.00
Sony CMD Z5	Frame	Smartphone	18.25	Polycarbonate	0.48	0.00	1190.00	4.00	0.00	0.00	0.00
Samsung GTI3200	Frame	Smartphone	16.09	Other polymers	0.00	0.00	7460.00	4.00	0.00	0.00	0.00
Samsung Gti 9300	Frame	Smartphone	23.49	Other polymers	0.66	0.00	906.00	4.00	61.00	0.00	0.00
Samsung sgh e700	Frame	Smartphone	10.31	Other polymers	0.00	0.00	1510.00	5.00	36.00	0.00	0.00
Samsung GTC3500	Frame	Smartphone	23.03	Other polymers	0.00	0.00	829.00	5.00	54.00	0.00	0.00
Samsung J120 F Galaxy	Frame	Smartphone	14.38	Other polymers	1.43	0.00	975.00	6.00	0.00	0.00	0.00
Samsung GT55230	Frame	Smartphone	21.05	Other polymers	0.39	0.00	1480.00	7.00	65.00	0.00	0.00
Samsung sghe530	Frame	Smartphone	13.84	Other polymers	0.00	0.00	1770.00	9.00	14.00	0.00	0.00
Sony ericsson z1010	Frame	Smartphone	15.36	Other polymers	3.14	0.00	5600.00	11.00	18.00	0.00	0.00
Samsung J120 F Galaxy	Screen	Smartphone	6.47	Poly(methyl methacrylate)	0.00	0.00	225.00	0.00	0.00	0.00	0.00
Samsung Gti9195	Screen	Smartphone	7.08	Poly(methyl methacrylate)	0.00	0.00	293.00	0.00	0.00	0.00	0.00
Siemens c62	Screen	Smartphone	8.21	Poly(methyl methacrylate)	0.00	0.00	315.00	0.00	0.00	0.00	0.00
Samsung GT55230	Screen	Smartphone	9.47	Poly(methyl methacrylate)	0.00	0.00	518.00	0.00	0.00	0.00	0.00
Wiko_Barry	Screen	Smartphone	14.96	Poly(methyl methacrylate)	1.13	0.00	761.00	0.00	0.00	0.00	0.00
Samsung GT53650	Screen	Smartphone	9.52	Poly(methyl methacrylate)	0.00	0.00	0.00	0.00	0.00	0.00	0.00
Samsung GTC3500	Screen	Smartphone	10.37	Poly(methyl methacrylate)	0.00	0.00	0.00	0.00	0.00	0.00	0.00
Samsung Gti 9300	Screen	Smartphone	10.57	Poly(methyl methacrylate)	0.00	0.00	0.00	0.00	0.00	0.00	0.00
Samsung sgh250	Screen	Smartphone	2.78	Poly(methyl methacrylate)	0.00	0.00	0.00	0.00	0.00	0.00	0.00
Samsung sghe530	Screen	Smartphone	6.23	Poly(methyl methacrylate)	0.00	0.00	0.00	0.00	0.00	0.00	0.00
Siemens A35	Screen	Smartphone	6.41	Poly(methyl methacrylate)	0.00	0.00	0.00	0.00	0.00	0.00	0.00
Siemens s25	Screen	Smartphone	10.32	Poly(methyl methacrylate)	0.00	0.00	0.00	0.00	0.00	0.00	0.00
Siemens A50	Screen	Smartphone	6.22	Poly(methyl methacrylate)	0.00	0.00	1050.00	0.00	0.00	0.00	0.00
Sony ericsson z1010	Screen	Smartphone	6.91	Polycarbonate	0.00	0.00	1670.00	0.00	0.00	0.00	0.00
Samsung SM 850	Screen	Smartphone	6.74	Poly(methyl methacrylate)	0.20	0.00	787.00	0.00	0.00	0.00	0.00

**Table A2:** Descriptive statistics of Br, Sb, Cl, Pb, Cr, Cd, and Hg concentrations and sample weights on the entire dataset.

Statistic	Br (mg/kg)	Sb (mg/kg)	Cl (mg/kg)	Pb (mg/kg)	Cr (mg/kg)	Cd (mg/kg)	Hg (mg/kg)	Sample Weight (g)
No. of observations	275	275	275	275	275	275	275	275
Minimum	0.000	0.000	0.000	0.000	0.000	0.000	0.000	0.000
Maximum	0.202	0.000	4.070	0.009	0.092	0.000	0.002	80.414
1st Quartile	0.000	0.000	0.000	0.000	0.000	0.000	0.000	7.738
Median	0.000	0.000	0.037	0.000	0.000	0.000	0.000	10.727
3rd Quartile	0.000	0.000	0.144	0.000	0.000	0.000	0.000	14.300
Mean	0.001	0.000	0.175	0.000	0.002	0.000	0.000	11.534
Variance	0.000	0.000	0.231	0.000	0.000	0.000	0.000	56.201
Standard deviation	0.013	0.000	0.480	0.001	0.009	0.000	0.000	7.497

**Table A3:** Descriptive statistics of Br, Sb, Cl, Pb, Cr, Cd, and Hg concentrations and sample weights on the “feature phones” set.

Statistic	Br (mg/kg)	Sb (mg/kg)	Cl (mg/kg)	Pb (mg/kg)	Cr (mg/kg)	Cd (mg/kg)	Hg (mg/kg)	Sample Weight (g)
No. of observations	208	208	208	208	208	208	208	208
Minimum	0.000	0.000	0.000	0.000	0.000	0.000	0.000	0.699
Maximum	0.202	0.000	3.260	0.009	0.092	0.000	0.002	30.316
1st Quartile	0.000	0.000	0.000	0.000	0.000	0.000	0.000	8.616
Median	0.000	0.000	0.037	0.000	0.000	0.000	0.000	10.888
3rd Quartile	0.000	0.000	0.151	0.000	0.000	0.000	0.000	14.079
Mean	0.002	0.000	0.155	0.000	0.002	0.000	0.000	11.491
Variance	0.000	0.000	0.124	0.000	0.000	0.000	0.000	25.126
Standard deviation	0.015	0.000	0.352	0.001	0.010	0.000	0.000	5.013

**Table A4:** Descriptive statistics of Br, Sb, Cl, Pb, Cr, Cd, and Hg concentrations and sample weights on the “smartphones” set.

Statistic	Br (mg/kg)	Sb (mg/kg)	Cl (mg/kg)	Pb (mg/kg)	Cr (mg/kg)	Cd (mg/kg)	Hg (mg/kg)	Sample Weight (g)
No. of observations	54	54	54	54	54	54	54	54
Minimum	0.000	0.000	0.000	0.000	0.000	0.000	0.000	0.702
Maximum	0.018	0.000	4.070	0.001	0.010	0.000	0.000	80.414
1st Quartile	0.000	0.000	0.000	0.000	0.000	0.000	0.000	7.082
Median	0.000	0.000	0.065	0.000	0.000	0.000	0.000	12.090
3rd Quartile	0.000	0.000	0.147	0.000	0.000	0.000	0.000	16.006
Mean	0.000	0.000	0.236	0.000	0.001	0.000	0.000	14.377
Variance	0.000	0.000	0.518	0.000	0.000	0.000	0.000	151.488
Standard deviation	0.003	0.000	0.719	0.000	0.002	0.000	0.000	12.308

**Table A5:** Descriptive statistics of Br, Sb, Cl, Pb, Cr, Cd, and Hg concentrations and sample weights on the “casings” components.

Statistic	Br (mg/kg)	Sb (mg/kg)	Cl (mg/kg)	Pb (mg/kg)	Cr (mg/kg)	Cd (mg/kg)	Hg (mg/kg)	Sample Weight (g)
No. of observations	90	90	90	90	90	90	90	90
Minimum	0.000	0.000	0.000	0.000	0.000	0.000	0.000	0.699
Maximum	0.202	0.000	4.070	0.005	0.092	0.000	0.000	80.414
1st Quartile	0.000	0.000	0.000	0.000	0.000	0.000	0.000	5.170
Median	0.000	0.000	0.102	0.000	0.000	0.000	0.000	8.880
3rd Quartile	0.000	0.000	0.211	0.000	0.000	0.000	0.000	12.645
Mean	0.003	0.000	0.265	0.000	0.005	0.000	0.000	10.766
Variance	0.000	0.000	0.382	0.000	0.000	0.000	0.000	111.850
Standard deviation	0.022	0.000	0.618	0.001	0.014	0.000	0.000	10.576

**Table A6:** Descriptive statistics of Br, Sb, Cl, Pb, Cr, Cd, and Hg concentrations and sample weights on the “frames” components.

Statistic	Br (mg/kg)	Sb (mg/kg)	Cl (mg/kg)	Pb (mg/kg)	Cr (mg/kg)	Cd (mg/kg)	Hg (mg/kg)	Sample Weight (g)
No. of observations	84	84	84	84	84	84	84	84
Minimum	0.000	0.000	0.000	0.000	0.000	0.000	0.000	4.560
Maximum	0.037	0.000	2.220	0.009	0.056	0.000	0.002	30.316
1st Quartile	0.000	0.000	0.000	0.000	0.000	0.000	0.000	11.816
Median	0.000	0.000	0.033	0.000	0.000	0.000	0.000	14.141
3rd Quartile	0.000	0.000	0.141	0.000	0.000	0.000	0.000	18.600
Mean	0.001	0.000	0.126	0.000	0.002	0.000	0.000	15.059
Variance	0.000	0.000	0.079	0.000	0.000	0.000	0.000	19.218
Standard deviation	0.005	0.000	0.281	0.001	0.006	0.000	0.000	4.384

**Table A7:** Descriptive statistics of Br, Sb, Cl, Pb, Cr, Cd, and Hg concentrations and sample weights on the “frames” components.

Statistic	Br (mg/kg)	Sb (mg/kg)	Cl (mg/kg)	Pb (mg/kg)	Cr (mg/kg)	Cd (mg/kg)	Hg (mg/kg)	Sample Weight (g)
No. of observations	88	88	88	88	88	88	88	88
Minimum	0.000	0.000	0.000	0.000	0.000	0.000	0.000	2.782
Maximum	0.011	0.000	3.260	0.000	0.000	0.000	0.000	22.737
1st Quartile	0.000	0.000	0.000	0.000	0.000	0.000	0.000	8.401
Median	0.000	0.000	0.030	0.000	0.000	0.000	0.000	10.267
3rd Quartile	0.000	0.000	0.082	0.000	0.000	0.000	0.000	13.194
Mean	0.000	0.000	0.155	0.000	0.000	0.000	0.000	10.658
Variance	0.000	0.000	0.245	0.000	0.000	0.000	0.000	10.981
Standard deviation	0.001	0.000	0.495	0.000	0.000	0.000	0.000	3.314

**Table A8:** Descriptive statistics of Br, Sb, Cl, Pb, Cr, Cd, and Hg concentrations and sample weights on the “polycarbonate” fraction.

Statistic	Br (mg/kg)	Sb (mg/kg)	Cl (mg/kg)	Pb (mg/kg)	Cr (mg/kg)	Cd (mg/kg)	Hg (mg/kg)	Sample Weight (g)
No. of observations	147	147	147	147	147	147	147	147
Minimum	0.000	0.000	0.000	0.000	0.000	0.000	0.000	0.699
Maximum	0.202	0.000	4.070	0.009	0.061	0.000	0.000	80.414
1st Quartile	0.000	0.000	0.000	0.000	0.000	0.000	0.000	8.845
Median	0.000	0.000	0.062	0.000	0.000	0.000	0.000	12.065
3rd Quartile	0.000	0.000	0.166	0.000	0.000	0.000	0.000	15.075
Mean	0.002	0.000	0.202	0.000	0.003	0.000	0.000	12.967
Variance	0.000	0.000	0.297	0.000	0.000	0.000	0.000	76.328
Standard deviation	0.017	0.000	0.545	0.001	0.010	0.000	0.000	8.737

**Table A9:** Descriptive statistics of Br, Sb, Cl, Pb, Cr, Cd, and Hg concentrations and sample weights on the “polymethylmethacrylate” fraction.

Statistic	Br (mg/kg)	Sb (mg/kg)	Cl (mg/kg)	Pb (mg/kg)	Cr (mg/kg)	Cd (mg/kg)	Hg (mg/kg)	Sample Weight (g)
No. of observations	87	87	87	87	87	87	87	87
Minimum	0.000	0.000	0.000	0.000	0.000	0.000	0.000	2.782
Maximum	0.014	0.000	3.030	0.001	0.092	0.000	0.000	22.737
1st Quartile	0.000	0.000	0.000	0.000	0.000	0.000	0.000	8.423
Median	0.000	0.000	0.025	0.000	0.000	0.000	0.000	10.217
3rd Quartile	0.000	0.000	0.095	0.000	0.000	0.000	0.000	12.428
Mean	0.000	0.000	0.139	0.000	0.001	0.000	0.000	10.438
Variance	0.000	0.000	0.159	0.000	0.000	0.000	0.000	10.579
Standard deviation	0.002	0.000	0.399	0.000	0.010	0.000	0.000	3.253

**Table A10:** Descriptive statistics of Br, Sb, Cl, Pb, Cr, Cd, and Hg concentrations and sample weights on the “other polymers” fraction.

Statistic	Br (mg/kg)	Sb (mg/kg)	Cl (mg/kg)	Pb (mg/kg)	Cr (mg/kg)	Cd (mg/kg)	Hg (mg/kg)	Sample Weight (g)
No. of observations	28	28	28	28	28	28	28	28
Minimum	0.000	0.000	0.000	0.000	0.000	0.000	0.000	0.702
Maximum	0.000	0.000	2.220	0.003	0.014	0.000	0.002	24.246
1st Quartile	0.000	0.000	0.000	0.000	0.000	0.000	0.000	6.152
Median	0.000	0.000	0.098	0.000	0.000	0.000	0.000	13.733
3rd Quartile	0.000	0.000	0.169	0.000	0.002	0.000	0.000	16.298
Mean	0.000	0.000	0.227	0.000	0.002	0.000	0.000	12.767
Variance	0.000	0.000	0.201	0.000	0.000	0.000	0.000	43.217
Standard deviation	0.000	0.000	0.449	0.001	0.003	0.000	0.000	6.574
